# Announcing the *Ochsner Journal* Impact Factor

**DOI:** 10.31486/toj.23.5036

**Published:** 2023

**Authors:** Ronald G. Amedee

**Affiliations:** Director of Clinical School and Professor, The University of Queensland Medical School, Ochsner Clinical School, New Orleans, LA; Editor-in-Chief, *Ochsner Journal*

*Fall is a Southerner's reward for having survived the summer.*

–*James Farmer*

The Fall 2023 issue of the *Ochsner Journal* contains four original research articles, one innovative program report, a quality improvement article, and five case reports and clinical observations which serve as the principal elements for this edition. This issue also contains the third quarterly Health, Medicine, and Society column, this time authored by Deborah F. Grimes, the Ochsner Health Chief Diversity Officer.

The original research includes an article by Pettus, Gavlinski, Beermann, et al on “Implementation of and Barriers to Optimizing Postpartum Care by Resident and Attending Physicians” followed by “Prognostic Factors of Morbimortality in Patients Treated for Xanthogranulomatous Pyelonephritis” authored by Garcia-Chairez, Robles-Torres, Rios-Palacios, et al. Next, please find work by Price-Haywood and Burton detailing “Racial Differences in Strength of Associations Between Colorectal Cancer Screening, Area Deprivation, Demographics, and Clinical Characteristics” followed by “Is It All About the Form? Norm- vs Criterion-Referenced Ratings and Faculty Inter-Rater Reliability” authored by Scielzo, Abdelfattah, and Ryder.

An innovative program description is offered by a group of Ochsner physicians and trainees including Fixler, Oliaro, Frieden, et al who report on a very timely experience with “Alert to Action: Implementing Artificial Intelligence–Driven Clinical Decision Support Tools for Sepsis.” Our quality improvement article this edition comes from Riopelle, Kozmenko, Wyche, et al looking at “Lower Double-Wall Puncture Rate During Ultrasound-Guided Internal Jugular Vein Cannulation Using Sharper, Narrower-Gauge, and/or Length-Optimized Needles: A 6-Year Quality Improvement Clinical Series in Adult Patients.”

Case reports and clinical observations include “Carcinosarcoma of the Esophagus–A Diagnostic Challenge” authored by Jain, Varshney, Aggarwal, et al, followed by “Syncope and Cardiac Arrhythmias Caused by a Paratracheal Mass” authored by Clayton, Eckholdt, Samra, et al. Jain, Selvakumar, Varshney, et al contributed “Gangliocytic Paraganglioma of the Duodenum: A Masquerader,” and Kajy, Rechenberg, Kerndt, and Wolschleger discuss “Cardiac Tamponade Secondary to Hemorrhagic Pericardial Effusion: A Complication of STEMI.” Rounding out this section is “Massive Renal Cyst Displacing Intra-Abdominal Structures” by Mohammed, Zarm, Velez, and Mohamed.

Summer 2023 in the Deep South will certainly go down in the record books as one of the, if not **the**, hottest summers temperature-wise since weather records were first kept. The promise of cooler temperatures is not far away, and Southerners will certainly welcome them as a reward for surviving this summer.

*Ochsner Journal* accomplished a major milestone this summer: for the first time ever, the *Journal* has been assigned a journal impact factor (JIF) in Clarivate Analytics Journal Citation Reports. For 2022, the *Ochsner Journal* JIF is 1.2. In addition to a solid JIF, the *Ochsner Journal* offers several publication benefits: full-text availability at PubMed, Web of Science, Google Scholar, and other databases; open-access publication with absolutely no article processing charges or other publication fees (compared to the thousands of dollars charged by other journals); and CC BY copyright that vests copyright with the authors, not with the *Journal*.

Since its inception in 1999, the *Ochsner Journal* has been a pioneer in nonpredatory, open-access publishing. Congratulations to Managing Editor Kathleen McFadden, the editorial board, our reviewers, and all who have trusted their work to our review and publication for making this distinction possible.

